# Risperidone and aggression in autism: A systematic review and meta-analysis

**DOI:** 10.1192/j.eurpsy.2026.10578

**Published:** 2026-07-31

**Authors:** N. Almutairi, R. Short, H. Gadelrab, B. Oakley, D. Murphy, M. M. Petrinovic

**Affiliations:** 1 Department of Forensic and Neurodevelopmental Sciences, Institute of Psychiatry, Psychology, and Neuroscience, King’s College London, London, United Kingdom; 2 Department of Psychology, Sabah Al Salem University City, Kuwait University, Kuwait City, Kuwait

## Abstract

**Introduction:**

Aggressive behaviours affect approximately two-thirds of autistic individuals, with significant consequences for their own quality of life and that of their families. Risperidone is among the most frequently prescribed medications for managing aggression in autism. However, prior systematic reviews have pooled heterogeneous study designs, limiting the strength of their conclusions.

**Objectives:**

This systematic review and meta-analysis synthesises evidence from randomised controlled trials (RCTs) on risperidone’s efficacy in reducing aggression in autism and its safety profile.

**Methods:**

Five databases (Embase, Ovid Medline, PsycInfo, Global Health, and PubMed) were systematically searched from inception to 2025. Eligible studies were randomised controlled trials (RCTs) assessing risperidone for the treatment of aggression in autistic children, adolescents, or adults. Risk of bias was evaluated using the Cochrane RoB tool. Random-effects models with standardised mean differences (SMDs) were applied to compare risperidone with placebo.

**Results:**

Thirteen studies met the inclusion criteria for the systematic review; eight RCTs (N = 445; age range 2.5–56 years) were included in the meta-analysis. Risperidone was significantly more effective than placebo in reducing aggression, with a large effect size (SMD = –0.96, 95% CI [–1.37, –0.55], Z = 4.62, p < .00001). Substantial heterogeneity was observed (I² = 73%). The most frequently reported adverse effects were weight gain, increased appetite, sedation, somnolence, and fatigue.

**Conclusions:**

Risperidone reduces aggression in autistic individuals but is associated with adverse side effects that restrict its long-term clinical use. Further research is needed to evaluate safer pharmacological alternatives and to develop more targeted treatments for aggression in autism.

**Disclosure of Interest:**

None Declared

